# Special Section Guest Editorial: Polarimetry in Biomedical Optics

**DOI:** 10.1117/1.JBO.29.5.052901

**Published:** 2024-05-30

**Authors:** Alex Vitkin, Jessica C. Ramella-Roman, Nirmalya Ghosh

**Affiliations:** a University of Toronto, Department of Medical Biophysics, Toronto, Ontario, Canada; b University of Toronto, Department of Radiation Oncology, Toronto, Ontario, Canada; c University Health Network, Toronto, Ontario, Canada; d Princess Margaret Cancer Centre, Toronto, Ontario, Canada; e Florida International University, Miami, Florida, United States; f Indian Institute of Science Education and Research (IISER), Department of Physical Sciences and Centre of Excellence in Space Sciences India (CESSI), Kolkata, West Bengal, India

## Abstract

The editorial introduces the two-issue JBO Special Section on Polarimetry in Biomedical Optics and provides resources for further exploration.

We are pleased to introduce new developments and state-of-the-art research in the exciting field of biophotonic polarimetry in this JBO Special Section entitled “Polarimetry in Biomedical Optics.” Due to the encouragingly large number of high-quality submissions, we have split the Special Section into two issues, one published in October 2023 and the other in May 2024. The issues cover various topics—from the mathematical and statistical characterization of polarimetric signals, to the development of novel instrumentation, the integration of AI and machine learning (ML) in analyzing polarization-sensitive data, and diverse medical applications. This impressive variety of enabling technology approaches and (pre)clinical applications speaks to the exciting biomedical potential (in some instances realized!) of value-added contrast afforded by the polarization properties of light towards a sensitive tissue assessment tool.

In the emerging field of polarization microscopy and digital pathology space, several authors have shown how digital pathology can be enhanced through polarization and AI. Majumdar et al. (https://doi.org/10.1117/1.JBO.29.5.052915) showed increased prognostic value of peri-tumoral collagen in colorectal cancer using Mueller matrix polarized light microscopy combined with ML, potentially improving personalized therapy selection by predicting 5-year local recurrence with high accuracy. Pham et al. (https://doi.org/10.1117/1.JBO.29.5.052917) focused on breast cancer imaging with polarized light imaging of various human pathologies, demonstrating its potential as an assistive tool for breast cancer diagnosis and classification. Sdobnov et al. (https://doi.org/10.1117/1.JBO.28.10.102903) proposed an enhanced Mueller-matrix polarimetry method that integrates wavelet decomposition and polarization-singular processing, to efficiently diagnose local tissue abnormalities in prostate histological sections. Robinson et al. (https://doi.org/10.1117/1.JBO.28.10.102904) integrated wide-field imaging Mueller polarimetry with ML for automated diagnostic segmentation of cervical tissue specimens, highlighting the importance of employing conservative classifier training strategies to mitigate bias and enhance accuracy in detecting pre-cancerous conditions. Deng et al. (https://doi.org/10.1117/1.JBO.28.10.102909) investigated the influence of the prevalent histological staining method that uses hematoxylin and eosin (H&E), on linear birefringence measurement of fibrous tissue structures; they report that while staining enhances linear retardance values, it does not significantly alter imaging contrast, suggesting the viability of using H&E stained tissue slices for clinical polarimetry applications. These representative examples nicely illustrate the strengths of polarimetric microscopy enhancements—rich biophysical information content contained in the various obtained polarization metrics, relatively simple instrumentation (often directly integratable into existing imaging systems), wide-field imaging capabilities, fast and robust measurements, ability to generate large data sets that may enable further AI-assisted analysis, and so on.

Novel polarimetric instrumentation research is represented in this Special Section by the study of Harper et al. (https://doi.org/10.1117/1.JBO.28.10.102910; featured on the cover of the October 2023 issue) who proposed a Doppler-tracked polarization-sensitive optical coherence tomography system for investigating the porcine spine. Boonya-Ananta et al. (https://doi.org/10.1117/1.JBO.29.5.052918) showed a novel speculum-free portable imaging system that characterizes collagen in the uterine cervix.

Finally, several papers focused on the fundamentals of polarized light transport in biological media. Lupushenko et al. (https://doi.org/10.1117/1.JBO.29.5.052913) explored the evolution of circularly polarized light in turbid media with Monte Carlo simulations and experimental validation. Bonaventura et al. (https://doi.org/10.1117/1.JBO.29.5.052914; featured on the cover of the May 2024 issue) studied the interaction of polarized light with fibers and fibrous tissue, including their inclination and curvature as a function of different imaging wavelengths, with promising findings suggesting the potential for accurate microstructural mapping of neural tissue. Kumar et al. (https://doi.org/10.1117/1.JBO.29.5.052916) elaborated on various decomposition methods when utilizing Mueller matrix imaging to study cervical cancer. Such examinations of the underlying science of polarized light–disordered media interactions lay an essential foundation for instrument design/optimization, biomedical application selection, and polarimetric results interpretation.

In conclusion, there are myriad innovative activities related to biomedical polarimetry, with new groups constantly entering this exciting field worldwide. We thus hope that the illustrative examples in this Special Section convey some of the novelty and excitement in this emerging field. Related resources include the SPIE Henri Poincaré Webinar Series (https://spie.org/conferences-and-exhibitions/spie-online/poincare-series), which is focused on the science and applications of polarized light (biomedical and beyond), as well as the JBO Hot Topics Webinar on Polarimetry in Biomedical Optics, moderated by Jessica Ramella-Roman, with presentations from Alex Vitkin, Tatiana Novikova, and Nirmalya Ghosh (archived here: https://www.spiedigitallibrary.org/jbo-hot-topics-webinar-series). Furthermore, in 2025, SPIE Photonics West is hosting a conference entitled “Polarized Light and Optical Momentum for Biomedical Applications” (San Francisco; https://spie.org/conferences-and-exhibitions/photonics-west). We urge interested readers to enjoy the articles in this two-part Special Section, look up pertinent references, and take advantage of additional resources available through SPIE and numerous other sources.

